# Clinical and economic effectiveness of telehealth in home mechanical ventilation

**DOI:** 10.1183/23120541.00283-2026

**Published:** 2026-09-28

**Authors:** Paula Johanna Filser, Pauline Sarah Münchenberg, Elke Berger, Reinhard Busse, Carmen Garcia, Tobias Kurth

**Affiliations:** 1 Institute of Public Health, Charité – Universitätsmedizin Berlin, Berlin, Germany; 2 Department of Health Care Management, Technische Universität Berlin, Berlin, Germany; 3 Department of Respiratory Medicine and Critical Care Medicine, Charité – Universitätsmedizin Berlin, Berlin, Germany

## Abstract

**Background:**

The number of patients requiring home mechanical ventilation is rising, while in-person follow-up is increasingly burdensome for patients and health systems. Telehealth may maintain clinical stability and improve care efficiency. This systematic review examined the clinical and economic effectiveness of telehealth in the management of home mechanical ventilation.

**Methods:**

The study protocol was registered *a priori* in PROSPERO. A systematic search was conducted in MEDLINE, Embase and Web of Science. The reporting of the systematic review was aligned with the Preferred Reporting Items for Systematic Reviews and Meta-Analyses (PRISMA) 2020 guidelines. We included quantitative studies comparing telehealth-supported home ventilation with standard care, reporting clinical or economic outcomes.

**Results:**

From 356 identified studies, 11 met the inclusion criteria: nine randomised controlled trials, one cohort study and one retrospective analysis, mostly conducted in Europe, with a total of 1458 participants. The study populations included patients with, *e.g.* COPD, amyotrophic lateral sclerosis and obesity hypoventilation syndrome. Interventions involved telehealth-supported home ventilation management. Across studies, telehealth-supported care achieved clinical outcomes comparable to those of standard care. Arterial carbon dioxide tension (*P*_aCO_2__) control and health-related quality of life were similar between groups, with one study reporting greater *P*_aCO_2__ reduction with telemonitoring. Evidence on the number of hospitalisations and exacerbations was mixed. Mortality and survival rates showed nonsignificant trends towards benefit; one cohort study reported improved first-year survival. Economic outcomes varied: home initiation reduced costs, while long-term follow-up results were heterogeneous.

**Conclusions:**

elehealth-supported home ventilation appears to be a comparable alternative to in-hospital management and often provides economic advantages. Future research should focus on standardised outcome measures, longer follow-up and health-system-specific cost-effectiveness analyses to guide sustainable implementation.

## Shareable abstract

**Telehealth-supported home mechanical ventilation provides clinical outcomes comparable to standard care and may reduce costs, particularly for home initiation. It represents an efficient alternative for delivering long-term ventilatory care.** https://bit.ly/3RPjUBw

## Introduction

Due to an ageing population and an increase in the prevalence of chronic respiratory diseases worldwide, the number of people requiring either invasive or noninvasive home mechanical ventilation (HMV) for chronic respiratory insufficiency is growing [1]. HMV is used to treat various conditions, including COPD, obesity hypoventilation syndrome (OHS) and neuromuscular diseases, such as amyotrophic lateral sclerosis (ALS) [2–4]. However, HMV requires close clinical follow-up to detect possible complications, adjust ventilator parameters and prevent acute exacerbations; this is why regular in-person visits are the current standard of care, but they can be highly burdensome for patients and their caregivers, particularly given the high dependency on technical support [5–7].

Telehealth is a term used interchangeably with telemedicine and encompasses the use of medical information exchanged from one site to another through electronic communication to improve patient health [8, 9]. Telehealth interventions in HMV, *i.e.* the use of technology to provide health information, monitoring and medical care remotely, can facilitate the continuous assessment of ventilator parameters and vital signs, while video consultations enable timely interventions to be carried out without the need for hospital visits, saving time and resources [4, 10, 11]. For patients receiving long-term HMV, telehealth-supported care may reduce treatment burden by reducing the need for hospital or clinic visits and enabling care delivery in the home environment [1, 2]. Continuous remote supervision and facilitated access to the care team can provide patients and caregivers with a greater sense of security and continuity of care, while telemonitoring of ventilator data and symptoms enables the early identification of clinical or technical issues, allowing for timely treatment adjustments without compromising ventilatory effectiveness [1, 4]. Furthermore, digitally delivered interventions can reduce the number of unplanned hospital admissions and improve patient safety [12]. Telehealth interventions also offer the potential to lower healthcare costs through scalability and affordability, while improving access to care, especially in remote areas, and fostering a more patient-centred approach to improve health and the quality of healthcare [4, 13]. Telehealth is particularly relevant for patients receiving HMV, given the growing demand for HMV and strained in-hospital resources for chronic care, as it offers the potential to enhance monitoring, optimise clinical management and reduce the burden of inpatient care, while enabling more responsive and individualised care and enhancing patients’ health-related quality of life (HRQoL) [1, 2].

A recent systematic review and meta-analysis found that telehealth-supported home oxygen therapy significantly reduced the number of hospital admissions and improved HRQoL for patients with COPD [14]. The evidence further suggests that, although telehealth interventions may incur higher initial costs, they are likely to result in long-term savings by reducing the number of hospital admissions [14]. Furthermore, a narrative review discussed telemonitoring in HMV; however, its focus was largely on available technologies, monitoring parameters and implementation challenges in HMV, rather than providing a structured synthesis of the evidence on the effectiveness of telehealth interventions in this area [1]. While these studies make important contributions, many questions remain unanswered, and further research is needed to fully understand the clinical and economic effectiveness of telehealth interventions in HMV.

Recently, an increasing number of randomised controlled trials (RCTs) have evaluated telehealth interventions in HMV. This systematic review aims to synthesise and critically appraise the existing evidence on their clinical effectiveness and cost-effectiveness, considering both patient-centred outcomes, including HRQoL, and system-level implications.

## Methods

### Protocol registration

Prior to conducting this systematic review, the study protocol was registered in the PROSPERO international prospective register of systematic reviews (PROSPERO CRD420251130435, https://www.crd.york.ac.uk/PROSPERO/view/CRD420251130435).

### Selection criteria

The aim of this study was to systematically identify, review and synthesise the existing evidence regarding the clinical and economic effectiveness of telehealth interventions among patients undergoing HMV.

### Inclusion and exclusion criteria

This review included studies involving patients of all ages who received HMV, either invasively or noninvasively, for any underlying medical condition requiring long-term ventilatory support. Eligible studies had to examine telehealth interventions directly related to ventilatory care, such as telemonitoring, remote patient monitoring, video consultations, mobile health applications or virtual care platforms. Studies were required to report clinical outcomes, such as hospitalisations, survival rates, mortality rates, exacerbations and HRQoL, and/or health economic outcomes, including healthcare costs and cost-effectiveness analyses. Only quantitative primary studies with standard care as a comparator were considered. Furthermore, included studies had to be peer-reviewed and available in full-text format.

Studies were excluded if they did not involve patients on mechanical ventilation, if they focused solely on inpatient or intensive care unit populations or if they evaluated telehealth interventions that were not related to ventilatory care. Studies focusing exclusively on technical aspects, such as device or circuit parameters, without reporting clinical or patient-centred outcomes, were also excluded. Studies lacking clinical or health economic outcome data, reporting only feasibility or satisfaction results, or with no comparator group were not considered. In addition, simulation studies, study protocols without published results, reviews, meta-analyses and other non-empirical publications (including editorials, commentaries, guidelines, dissertations and conference abstracts) were excluded from the analysis.

### Search strategy

A comprehensive and systematic literature search was conducted across three electronic databases (MEDLINE, Embase and Web of Science) to identify relevant studies published from database inception to 2 September 2025. The search was structured into three concept blocks: outcome with clinical outcomes and economic outcomes, which were combined with the “AND” operator; telehealth interventions; and HMV. All three blocks were combined using the “AND” operator. The search strategy combined keywords and Medical Subject Headings (MeSH) such as telehealth, telemedicine, home ventilation and clinical effectiveness. The Boolean operators “OR” and “AND” were used to appropriately link and refine the search terms across the title, abstract and keyword fields. The full search strategy is provided in table S1. In addition to the database searches, the reference lists from included studies and previous systematic reviews were manually screened to identify any additional eligible publications.

All retrieved records were imported into Rayyan, a web-based application for systematic reviews [15], where duplicate entries were identified and manually removed. Subsequently, a two-step screening process was performed. First, titles and abstracts were screened for relevance. Second, the full texts of potentially eligible studies were reviewed in detail, followed by data extraction from studies meeting the inclusion criteria. The following data were extracted: 1) study characteristics (*e.g.* authors, year of publication, country), 2) type of ventilation and underlying disease, 3) characteristics of the telehealth intervention (*e.g.* intervention type and provider), 4) clinical effectiveness outcomes and 5) economic effectiveness outcomes. The collected data were compiled in a standardised Excel sheet. To prevent duplication, studies originating from the same institution were cross-checked to ensure that each publication represented a distinct dataset.

The screening and data extraction processes were conducted independently by two reviewers (PJF and PSM). Any discrepancies were resolved through discussion, and, if necessary, a third reviewer (EB) was consulted to reach a consensus on study inclusion and extracted information. The systematic review is reported in accordance with Preferred Reporting Items for Systematic Reviews and Meta-Analyses (PRISMA) 2020 reporting guidelines [16], which are listed in table S2.

### Risk of bias

The risk of bias in the included studies was evaluated using validated assessment tools appropriate to each study design. RCTs were appraised with the Cochrane Risk-of-Bias 2 (RoB 2) tool [17], while non-randomised studies were assessed using the Risk Of Bias In Non-randomized Studies – of Interventions, version 2 (ROBINS-I V2), instrument [18]. To enable a coherent narrative summary, the domain-specific judgements from ROBINS-I were mapped to overall risk levels using RoB 2 terminology (low risk of bias, some concerns, high risk of bias), as the two assessment tools use slightly different rating categories and thresholds. Accordingly, ROBINS-I ratings of low risk were classified as having a low risk of bias, moderate risk as some concerns and serious or critical risk as high risk of bias [17, 18]. Two reviewers (PJF and PSM) independently assessed each study for risk of bias. Any disagreements were resolved through discussion and, if needed, consultation with a third reviewer (EB) to reach consensus.

## Results

### Study selection and data extraction

The literature search identified 356 records, of which 154 were duplicates and so were removed. PJF and PSM screened the remaining 202 abstracts, excluding a further 187 that did not meet the predefined inclusion and exclusion criteria. 15 articles were selected for full-text assessment, resulting in the exclusion of a further four studies. Consequently, 11 studies were included in the final analysis. Figure 1 shows the detailed search and selection process in accordance with the PRISMA flow diagram.

**FIGURE 1:**
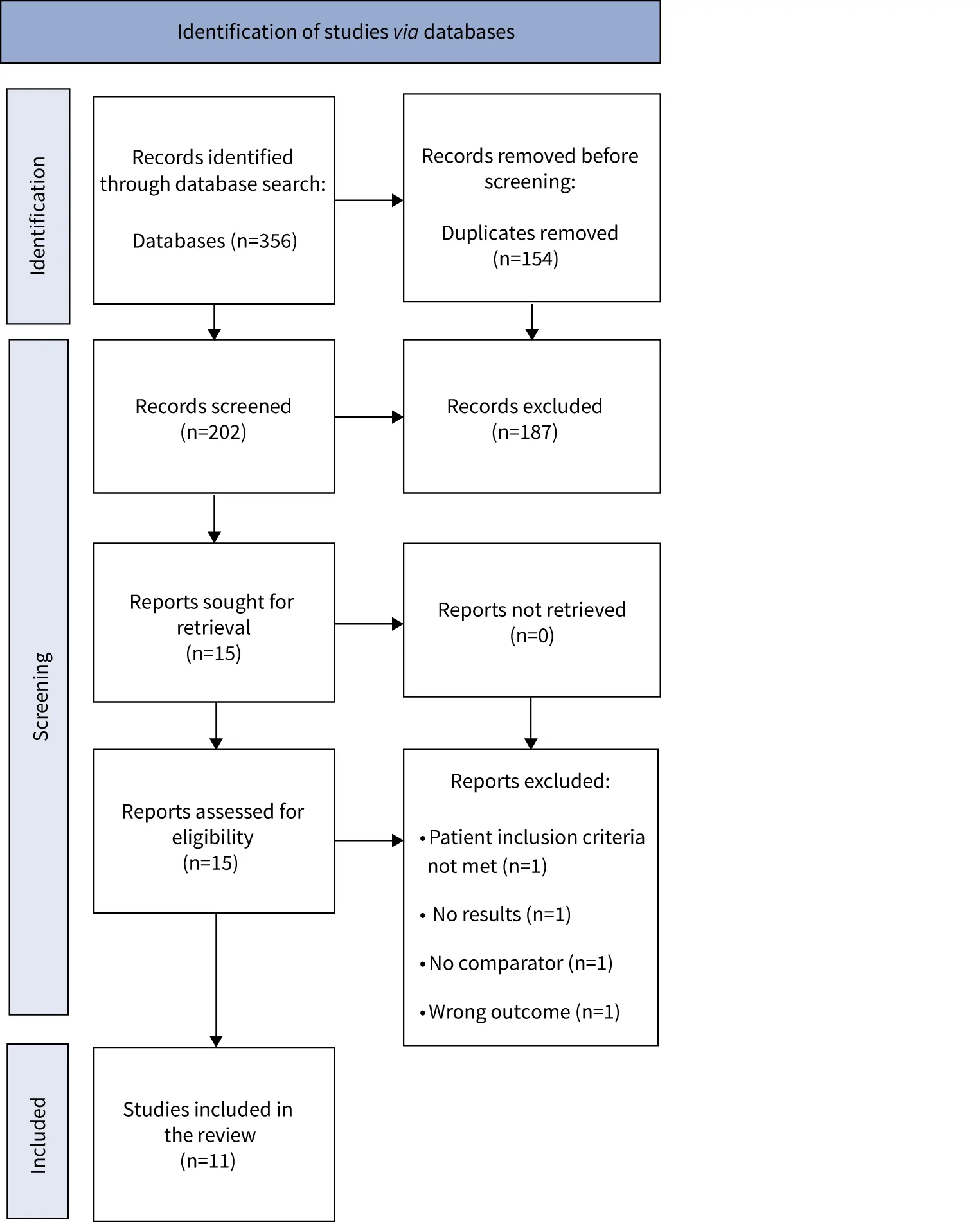
Flow chart of study identification, screening and selection according to the Preferred Reporting Items for Systematic Reviews and Meta-Analyses (PRISMA) 2020 guidelines [16].

### Study characteristics

The characteristics of the included studies are summarised in table 1.

**TABLE 1:** Characteristics of studies evaluating telehealth in home mechanical ventilation (HMV)

First author [Ref.], year	Country	Sample size (n)	Study type	Underlying disease	Primary study aim	Type of ventilation	Initiation setting	Type of intervention	Follow-up duration
**Vitacca [19], 2009**	Italy	220	RCT	COPD, NMD, ALS or other	Evaluate reduction in number of hospitalisations with TM for chronic respiratory patients	IV and NIV	Hospital	Tele-assistance	12 months
**Sheers [20], 2024**	Australia	23	RCT	Motor neurone disease (mostly ALS)	Compare an in-home NIV initiation model with remote monitoring *versus* usual care	NIV	Home	Home initiation and remote monitoring	12 weeks
**Van den Biggelaar [21], 2020**	Netherlands	96	RCT	NMD (mostly ALS) or thoracic cage disorder	Assess whether home-based HMV initiation with TM is non-inferior to in-hospital initiation nationwide	NIV	Home	Home initiation and remote monitoring	6 months
**Prigent [22], 2025**	France	53	RCT	COPD, CHRF	Assess the impact of TM compared with usual follow-up on home NIV effectiveness	NIV	Hospital	TM	6 and 12 months
**Pinto [23], 2010**	Portugal	39	RCT	ALS	Test the efficacy and efficiency of TM for NIV	NIV	Hospital	TM	Every 3 months for 3 years
**Jiang [24], 2024**	China	148	RCT	COPD	Assess the benefit of IoT-based management of home noninvasive positive pressure ventilation	NIV	Hospital	TM	12 months
**Hazenberg [25], 2014**	Netherlands	77	RCT	NMD or thoracic cage disorder	Assess whether home-based HMV initiation with TM is non-inferior to hospital initiation in selected patients with chronic respiratory failure	NIV	Home	Home initiation and remote monitoring	6 months
**Duiverman [26], 2019**	Netherlands	67	RCT	COPD or CHRF	Assess whether home-based NIV initiation with TM is non-inferior to in-hospital initiation in COPD patients	NIV	Home	Home initiation and remote monitoring	3 and 6 months
**Lopes de Almeida [27], 2012**	Portugal	39	RCT	ALS	Assess hospital and NHS costs associated with TM intervention	NIV	Hospital	TM	Every 3 months for 3 years
**Le Mao [28], 2024**	France	659	Cohort study	COPD, OHS or other	Assess whether TM reduces NIV dropout compared with a non-TM pathway	NIV	Hospital	TM	12 months
**Vitacca [29], 2016**	Italy	37	Retrospective analysis	COPD	Assess the impact of long-term care, including TM, on healthcare utilisation in COPD patients on long-term oxygen therapy	NIV	Hospital	Tele-assistance	12 months

TM: telemonitoring; NIV: noninvasive ventilation; RCT: randomised controlled trial; NMD: neuromuscular disease; ALS: amyotrophic lateral sclerosis; IV: invasive ventilation; CHRF: chronic hypercapnic respiratory failure; OHS: obesity hypoventilation syndrome; IoT: internet of things; NHS: National Health Service.

Of the 11 studies included, nine were RCTs [19–27], one was a cohort study [28] and one was a retrospective secondary analysis of an RCT [29]. In all studies, patients in the control group (CG) received standard inpatient care, whereas those in the intervention group (IG) received outpatient care *via* telehealth. Regarding ventilation initiation, four studies evaluated home-based HMV initiation supported by telehealth interventions [20, 21, 25, 26]. In contrast, the remaining studies investigated telehealth interventions implemented after in-hospital initiation, primarily during long-term follow-up [19, 22–24, 27, 29]. Studies were classified into three categories based on the type of telehealth intervention. Two studies investigated tele-assistance, a nurse-led model providing continuous remote clinical support with pulse oximetry monitoring [19, 29]. Five studies employed telemonitoring [22–24, 27], defined as the periodic remote transmission and monitoring of physiological data [30]. The remaining four studies evaluated home-based noninvasive ventilation (NIV) initiation combined with structured remote monitoring [20, 21, 25, 26]. The follow-up duration varied substantially across studies, ranging from 12 weeks to 12 months; two studies reported long-term follow-up of up to 3 years, with repeated assessments at regular intervals [23, 27]. All but one study included patients who received NIV only. The underlying diseases varied, with COPD being the most common [19, 22, 24, 26, 28, 29], followed by ALS or other neuromuscular diseases [19, 21, 23, 27]. Nine of the 11 studies were conducted in Europe [19, 21–23, 25–29], one in Australia [20] and one in China [24]. The included studies were published between 2009 and 2025. Sample sizes ranged from 23 to 659 participants.

### Principal findings

The primary outcomes of interest are presented in table 2 and table 3; table 4 presents the secondary outcomes.

**TABLE 2:** Clinical outcomes of the included studies: arterial carbon dioxide tension (*P*_aCO_2__) and hospitalisation and exacerbation rates

First author [Ref.], year	*P*_aCO_2__	Hospitalisation	Exacerbation
**Vitacca [19], 2009**	–	The number of hospitalisations per month was **significantly (p<0.01)** lower in the IG	IG group is more likely to remain free from acute exacerbations. Mean number of exacerbations per month was **significantly** higher in the CG
**Van den Biggelaar [21], 2020**	The adjusted mean difference between groups was 0.01 kPa	–	–
**Prigent [22], 2025**	*P*_aCO_2__ at 6 months was **significantly** lower in the IG than in the CG **(p=0.003)**	Fewer patients in the IG than in the CG were hospitalised	The number and severity of COPD exacerbations were similar between the IG and CG
**Jiang [24], 2024**	The CG and IG, daytime *P*_aCO_2__ decreased **significantly** over the follow-up. The adjusted mean between-group difference in change at 12 months was 0.47 mmHg	–	–
**Hazenberg [25], 2014**	*P*_aCO_2__ improved by 0.72 kPa (se 0.16 kPa) in the CG and by 0.91 kPa (se 0.20 kPa) in the IG	–	–
**Duiverman [26], 2019**	The mean between-group difference in change at 6 months (IG *versus* CG) was 0.04 kPa	No differences	No differences
**Vitacca [29], 2016**	The CG showed slightly higher mean values	Hospitalisation rates were 5% lower in the IG. Mean number of hospitalisations per patient per year was slightly lower in the IG	Fewer IG patients experienced ≥1 exacerbation. The IG had a **significantly** reduced exacerbation risk, compared with the CG

Unless stated otherwise, differences between groups were not statistically significant. CG: control group; IG: intervention group.

**TABLE 3:** Clinical outcomes of the included studies: health-related quality of life (HRQoL) and mortality and survival rates

First author [Ref.], year	HRQoL	Mortality	Survival
**Vitacca [19], 2009**	–	Mortality was higher in the CG	–
**Sheers [20], 2024**	No differences	–	–
**Van den Biggelaar [21], 2020**	No differences	–	–
**Pinto [23], 2010**	–	–	Patients in the CG tended to have longer survival
**Jiang [24], 2024**	Statistically **significant** differences in HRQoL between the IG and CG at 6 months, with greater improvements in the IG	–	–
**Hazenberg [25], 2014**	Improvements in SRI scores were non-inferior in the IG, compared with the CG. The MRF-28, HADS and SF-36 showed no differences	–	–
**Duiverman [26], 2019**	No differences	–	–
**Le Mao [28], 2024**	–	–	During the first year of treatment, 82 patients died: 7% in the IG and 16% in the CG **(p<0.001)**
**Vitacca [29], 2016**	–	12-month mortality was lower in the IG	–

Unless stated otherwise, differences between groups were not statistically significant. IG: intervention group; CG: control group; SRI: Severe Respiratory Insufficiency Questionnaire; MRF-28: Maugeri Respiratory Failure Questionnaire; HADS: Hospital Anxiety and Depression Scale; SF-36: 36-item Short-Form Health Survey.

**TABLE 4:** Economic outcomes of telehealth-supported home mechanical ventilation (HMV) according to initiation setting

First author [Ref.], year	Economic costs
**Initiating NIV/IV at home**	
Van den Biggelaar [21], 2020	The cost analysis showed an average saving of EUR 3225 (95% CI EUR 4279–2107) per patient when ventilatory support was started at home rather than at the hospital
Hazenberg [25], 2014	Mean total costs per patient were EUR 726 in the IG and EUR 3913 in the CG, with a between-group difference of EUR 3187 (95% CI EUR −3643–2694)
Duiverman [26], 2019	Initiating NIV at home costs less than half as much (EUR 3768, 95% CI EUR 3546–4163) as hospital initiation (EUR 8537, 95% CI EUR 7540–9175)
**Initiating NIV/IV at the hospital**	
Vitacca [19], 2009	After deducting the costs of the tele-assistance programme, the mean total cost per patient was lower in the IG than in the CG (IG: EUR 8907±17 580; CG: EUR 14 728±28 694)
Jiang^#^ [24], 2024	Average total costs per patient were EUR 1141 (95% CI EUR 1080–1202) in the IG and EUR 893 (95% CI EUR 832–953) in the CG, resulting in a between-group difference of EUR 248 (95% CI EUR 162–334; p<0.001). The average number of QALYs was 0.453 (95% CI 0.389–0.516) for the IG and 0.443 (95% CI 0.380–0.506) for the CG. The ICER was EUR 24 982 per QALY (95% CI EUR 18 811–31 140)
Lopes de Almeida [27], 2012	During the study period, mean total hospital costs per patient were EUR 5733.70±4276.10 in the CG and EUR 15 791.50±14 360.80 in the IG (p=0.008). By contrast, mean annual costs per patient did not differ significantly between groups (EUR 8882.80±2718.50 in the CG *versus* EUR 9508.40±1325.20 in the IG; p=0.36)

NIV: noninvasive ventilation; IV: invasive ventilation; IG: intervention group; CG: control group; QALY: quality-adjusted life-year; ICER: incremental cost-effectiveness ratio. ^#^: The study was conducted in China and reported costs in yuan, which were converted to euros, based on the European Central Bank exchange rate [44].

### Clinical outcomes

#### Arterial carbon dioxide tension

The arterial carbon dioxide tension (*P*_aCO_2__) is a key physiological marker of ventilatory adequacy and is commonly used to evaluate the effectiveness of HMV, including when monitored or adjusted *via* telehealth [31]. Six of the 11 studies reported *P*_aCO_2__ outcomes [21, 22, 24–26, 29]. Across these studies, differences between the IG and CG were generally small and not statistically significant. In one study, the mean *P*_aCO_2__ values in the IG were slightly higher than in the CG, but the difference was not significant (p=0.080) [29]. Another study found an adjusted mean difference of 0.01 kPa (95% CI −0.36–0.38), indicating non-inferiority of the CG compared with the IG [21]. Similarly, in other trials, the adjusted mean between-group differences in *P*_aCO_2__ change were small and nonsignificant, *e*.*g*. 0.47 mmHg (95% CI −3.47–4.41 mmHg; p=0.812) and 0.04 kPa (95% CI −0.31–0.38 kPa) [24–26]. However, one study reported a significant reduction in *P*_aCO_2__ at 6 months in the IG compared with the CG (p=0.003) [22]. Overall, the studies consistently showed no clinically relevant or statistically significant differences in *P*_aCO_2__ between groups, except for the telemonitoring intervention, which demonstrated improved *P*_aCO_2__ control [22].

#### Hospitalisation

Hospitalisations, typically defined as all-cause or disease-specific hospital admissions and including length of stay, is a key indicator of healthcare utilisation and clinical instability in patients with chronic respiratory conditions [32]. Four studies reported hospitalisation outcomes [19, 22, 26, 29]. Some studies report fewer hospital admissions in the IG [19, 22, 29]. However, another study reports that changes in number of hospital days and hospitalisation frequency did not differ between groups [26]. Overall, the evidence on hospitalisation outcomes was inconsistent: some studies showed fewer admissions, while others showed no differences.

#### Exacerbation

Exacerbations, typically defined as acute worsening of respiratory symptoms necessitating additional treatment or hospitalisation, are an important indicator of clinical stability and disease control in patients receiving telehealth-supported home ventilation [33]. Four studies reported exacerbation-related outcomes [19, 22, 26, 29]. Two of these studies showed a significant reduction in exacerbation risk or frequency in the IG, compared with the CG [19, 29]. By contrast, two studies found no significant differences in exacerbation frequency or severity between groups [22, 26]. Overall, the evidence was limited and inconsistent, with half of the studies demonstrating a beneficial effect of the intervention on exacerbations.

#### Health-related quality of life

HRQoL reflects the perceived impact of chronic respiratory failure, HMV and associated care processes on patients’ physical, psychological and social well-being [34]. Five studies evaluated HRQoL [20, 21, 24–26] using various validated questionnaires, including the Severe Respiratory Insufficiency Questionnaire (SRI) [35], the Maugeri Respiratory Failure Questionnaire (MRF-28) [36], the 36-item Short-Form Health Survey (SF-36) [37], the Hospital Anxiety and Depression Scale (HADS) [38], the Clinical COPD Questionnaire [39], the Assessment of Quality of Life [40], the Epworth Sleepiness Scale [41] and the ALS Functional Rating Scale – Revised [42].

Across most studies, there were no significant differences in HRQoL between groups. One study reported greater improvement at 6 months in the CG than in the IG, although differences at 3 months were not significant [24]. Another study found that both groups improved on certain SRI subscales, with home initiation being non-inferior to hospital initiation. The MRF-28 showed no significant differences between groups. The SF-36 showed improvement in vitality in both groups, and HADS scores remained unchanged [25]. Overall, the results demonstrated that home initiation was non-inferior to hospital initiation for HRQoL, with similar patterns of improvement observed across different HRQoL instruments, while findings on hospitalisation were limited and inconsistent across studies.

#### Mortality

Mortality, defined as the proportion of patients who die within a specified time frame, is a critical clinical end point used to evaluate the efficacy of telehealth interventions in home-ventilated populations [34, 43]. Two studies reported on mortality outcomes [19, 29]. In one study, the 12-month mortality rate was lower in the IG (14%) than in the CG (25%) [29]. The other study found a similar trend, with lower mortality in the IG (18%) than in the CG (23%), although this difference was not statistically significant (p=0.241). A multivariate Cox model identified tele-assistance (hazard ratio (HR) 0.310; p=0.032) and lower *P*_aCO_2__ (HR 0.81; p=0.014) as protective factors [19]. Overall, both studies suggested a trend towards reduced mortality with tele-assistance, although there were no statistically significant group differences.

#### Survival

The survival rate indicates whether telehealth-supported HMV is associated with increased life expectancy in patients with chronic respiratory failure [34, 43]. Two studies reported survival outcomes [23, 28]. In one study, survival at the end of the follow-up period (after 176 days) was similar between the two groups, with 12 patients alive in each. However, differences did not reach statistical significance (p=0.13), although patients in the CG tended to survive for longer [23]. By contrast, the other study found significantly better survival during the first year of NIV treatment in the telemonitoring group (7%) than in the non-telemonitoring group (16%) (p<0.001) [28]. Overall, one study reported no significant difference in survival, whereas the other showed a significant benefit associated with telemonitoring.

### Secondary findings

#### Costs

Six studies reported economic outcomes [19, 21, 24–27]. All but one study reported costs in euros; the one study conducted in China reported costs in yuan [24], which were converted to euros based on the European Central Bank exchange rate [44]. When the studies were stratified according to the setting in which ventilation was initiated, three studies evaluated the economic impact of telehealth-supported home-based initiation of noninvasive HMV [21, 25, 26], and three studies assessed telehealth interventions implemented after in-hospital initiation, primarily during long-term management [19, 24, 27]. Studies investigating home-based initiation consistently reported substantial cost savings compared with standard in-hospital initiation. One analysis from a societal perspective found an average saving of EUR 3225 (95% CI EUR 4279–2107) per patient when ventilation was initiated at home rather than in hospital [21]. Another study found a mean total cost per patient of EUR 726 in the IG, compared with EUR 3913 in the CG (difference EUR 3 187, 95% CI −EUR 3643–2694) [25]. Similarly, a third study showed that initiation of NIV at home was less than half the cost of hospital initiation (EUR 3768 (95% CI EUR 3546–4163) *versus* EUR 8537 (95% CI EUR 7540–9175)) [26]. By contrast, studies in which telehealth interventions were applied following in-hospital initiation showed more heterogeneous economic results. In one study, after deducting tele-assistance programme expenses, the average total cost per patient was lower in the IG than in the CG (IG: EUR 8907±17 580; CG: EUR 14 728±28 694) [19]. Another study, conducted in China, reported average annual costs of EUR 1141 (95% CI EUR 1080–1202) for patients in the IG and EUR 893 (95% CI EUR 832–953) for patients in the CG, with an incremental cost-effectiveness ratio of EUR 24 982 per quality-adjusted life-year (95% CI EUR 18 811–31 140) [24]. Another trial reported a higher mean hospital cost for patients in the IG (EUR 15 791±14 360) than for those in the CG (EUR 5734±4276; p=0.008), although there was no significant difference in annual mean costs (EUR 9508±1325 *versus* EUR 8883±2719 for the IG and CG, respectively; p=0.36) [27]. Overall, the reported economic outcomes differed according to the initiation setting. Telehealth-supported home-based initiation was associated with lower costs, whereas telehealth interventions implemented after in-hospital initiation were mostly associated with higher costs than standard care.

### Risk of bias

Both physicians and patients were aware of group allocation, as blinding was not possible due to the nature of the intervention. Of the nine RCTs, five were assessed as having a low risk of bias [21, 22, 24–26], and two showed some concerns due to deviations from the intended interventions [19, 20]. However, data were available for nearly all participants, and deviations were mostly resolved or balanced between groups. Two RCTs were rated as having a high risk of bias because group allocation was based on participants’ place of residence, rather than true randomisation. Nevertheless, all other domains of the assessment tool were considered to be at low risk of bias, and the lack of randomisation did not appear to affect the outcomes [23]. Both retrospective studies were rated as having a moderate risk of bias, mainly due to the lack of randomisation and potential selection bias. However, the interventions were clearly defined, missing data and selective reporting were not considered major issues and outcome assessment was based on routinely collected or objective measures [28, 29]. Both risk-of-bias tables can be found in table S3 and figure S1.

## Discussion

This systematic review synthesised the available evidence on the clinical and economic effectiveness of telehealth interventions in HMV. Overall, telehealth-supported care, including telemonitoring and remote consultations, can be a feasible alternative to conventional hospital-based management, achieving largely comparable outcomes in terms of *P*_aCO_2__ control, exacerbation rates, HRQoL, mortality and survival.

However, the effects of telehealth were not uniform across studies or the outcomes of this review, and a more nuanced interpretation of the individual findings is warranted. Across most trials, *P*_aCO_2__ control was comparable between IGs and CGs, indicating that telehealth-supported management does not compromise ventilatory effectiveness. While one randomised trial reported a significantly greater reduction in *P*_aCO_2__ with telemonitoring, other studies demonstrated only small, nonsignificant between-group differences. This heterogeneity likely reflects differences in intervention intensity, such as monitoring frequency, and the extent to which transmitted data were actively used for remote adjustment of ventilator settings. Evidence regarding reductions in hospitalisations and exacerbations remains limited, as only a small number of studies assessed these outcomes and findings were inconsistent.

The findings of this review are consistent with the growing body of literature that supports the use of telehealth in the management of chronic respiratory conditions [10–12]. Previous studies have shown that remote monitoring enables early detection of clinical deterioration, facilitates timely therapeutic adjustments and may prevent acute exacerbations, which can improve patient safety and reduce the number of hospitalisations [1, 4, 10]. Furthermore, telehealth interventions can improve continuity of care and patient autonomy, particularly for individuals with restricted mobility or those living in remote areas [5, 6, 12]. These aspects are becoming increasingly relevant as chronic respiratory insufficiency becomes more prevalent and more people depend on long-term ventilatory support [1, 7, 45].

However, it is important to note that much of the available literature has focused on specific disease populations, most frequently patients with COPD or neuromuscular disorders, while evidence across the broader spectrum of HMV indications remains limited [1, 12, 14, 46, 47]. For instance, many prior analyses have examined telemonitoring primarily in COPD patient groups, in which telehealth-supported management was associated with reduced exacerbation rates and improved treatment adherence [14]. Similarly, studies of neuromuscular disorders have focused on feasibility and ventilator adherence rather than comprehensive clinical or economic outcomes [1]. Consequently, generalising the results of disease-specific studies to the heterogeneous HMV population should be approached with caution. By contrast, the present review encompasses diverse respiratory diseases, including COPD, OHS, ALS and restrictive thoracic disorders. Thus, it offers a more integrative perspective on the role of telehealth across diagnostic groups.

The interpretation of these findings aligns with current clinical guidance. The clinical practice recommendations of the German Respiratory Society for noninvasive and invasive ventilation emphasise structured follow-up and the integration of telemonitoring as essential components of high-quality long-term ventilation. This is particularly important for ensuring safety, adherence and the individualised adjustment of ventilator settings [46]. Similarly, the European Respiratory Society has highlighted that telemonitoring can effectively complement standard care when supported by the appropriate infrastructure, data protection and clinical governance [47, 48]. These recommendations echo international initiatives advocating for the systematic integration of digital health technologies to improve accessibility and efficiency in chronic respiratory management [10, 12].

From an economic standpoint, existing evidence suggests that telehealth-supported care may be cost-effective as it can reduce the number of unplanned hospital admissions and optimise outpatient management [14]. While initial implementation costs can be substantial, long-term analyses indicate potential savings through decreased healthcare utilisation and more efficient resource allocation [14].

Some limitations must be considered when interpreting the results. A key limitation of this review relates to the heterogeneity of the included telehealth interventions. As used throughout this review, “telehealth” serves as an umbrella term encompassing distinct intervention types, including telemonitoring, tele-assistance and home initiation with remote monitoring, which differ in their intensity and degree of active clinical involvement, ranging from continuous 24/7 tele-assistance to weekly modem-based telemonitoring and remote monitoring during home initiation. These differ considerably in monitoring intensity, clinical intervention scope and the professional groups involved, which limits the comparability of outcomes across studies. A structured narrative comparison suggests that more intensive, continuous models (*e.g.* tele-assistance) tend to be associated with reductions in exacerbation frequency and hospitalisations, whereas less intensive telemonitoring approaches primarily demonstrate non-inferiority to standard care. A formal subgroup analysis was not feasible due to the limited number of studies per intervention subtype and marked heterogeneity in outcome definitions. However, this distinction should be explicitly considered when interpreting findings.

Beyond intervention heterogeneity, the risk-of-bias assessment warrants consideration when interpreting the findings. While the principal findings regarding *P*_aCO_2__ control and HRQoL are largely derived from low-risk RCTs, the two high-risk RCTs and two moderate-risk retrospective studies contributed primarily to the evidence on tele-assistance and survival outcomes, which are already interpreted with caution. Although a formal sensitivity analysis was not feasible given the limited number of studies, a qualitative assessment suggests that excluding higher-risk studies would not materially alter the overall conclusions. Nevertheless, residual confounding cannot be excluded, and effect sizes for hospitalisations, exacerbations and economic outcomes should be interpreted accordingly.

The clinical heterogeneity across disease populations (COPD, ALS, OHS, neuromuscular diseases) constitutes a further important limitation. These conditions differ substantially in their disease trajectory, prognosis and ventilatory needs, which affects the generalisability of findings. The rationale for encompassing diverse disease entities reflects the aim of this review to assess telehealth effectiveness across the full clinical spectrum of HMV. Nevertheless, disease-specific conclusions should be drawn with caution, and disease-stratified analyses are recommended as a priority for future research, contingent on a sufficient accumulation of evidence per diagnostic group.

Outcome heterogeneity, particularly regarding HRQoL and exacerbations, represents a further limitation. Included studies employed a variety of measurement instruments and questionnaires, limiting direct comparability across studies. Importantly, however, this review focused primarily on between-group differences rather than absolute outcome values, assessing whether telehealth-supported care achieved comparable results to standard care. This approach mitigates some of the impact of outcome heterogeneity on the overall conclusions, as the direction and magnitude of group differences remain interpretable even when different instruments are used. Similarly, the economic analyses were intended to compare cost differences between telehealth and standard care within each study, rather than to report transferable absolute cost values. Nevertheless, even this within-study comparison is affected by substantial contextual differences. The included studies varied in their economic perspectives: one study explicitly adopted a societal perspective [21], some applied a healthcare system perspective [19, 24] and other studies did not clearly specify the perspective taken. Transferability across health systems is further limited by structural differences in reimbursement models, digital infrastructure and the professional roles involved in telehealth delivery. The lack of standardised outcomes and transparent reporting of economic perspectives limits the external validity of individual findings and precludes more granular synthesis. Future studies should ideally adhere to a standardised core outcome set, including *P*_aCO_2__ as a key physiological marker of ventilatory adequacy, HRQoL assessed with a validated disease-specific instrument such as the SRI, hospitalisation rates, exacerbation frequency and healthcare costs reported from a clearly defined economic perspective, to enable meaningful cross-study comparisons [1, 4, 49].

In addition, many of the included studies had small sample sizes and short follow-up periods. Blinding was not feasible, introducing possible performance bias. Although nine RCTs were included, a meta-analysis was not performed. This decision was based on specific methodological considerations: heterogeneity in patient populations, disease severity, ventilatory settings, different telehealth interventions, inconsistency in outcome definitions and measurement instruments and heterogeneity in follow-up duration and reported statistical measures, which preclude meaningful aggregation. A pooled effect estimate under these conditions would risk producing a spuriously precise result that obscures rather than clarifies the underlying evidence.

Despite these limitations, the review has several strengths. First, it provides a comprehensive synthesis of both clinical and economic dimensions of telehealth in HMV across multiple disease entities. Second, including studies with nonsignificant results reduces publication bias, and the consistently low attrition rates across studies highlight the feasibility and acceptance of telehealth among patients and caregivers. These findings are consistent with qualitative research in the field [6].

### Conclusion

The growing number of patients requiring long-term HMV has sparked increased interest in telehealth as a means of optimising healthcare and reducing the burden on hospitals. Telehealth-supported HMV appears clinically comparable to conventional in-hospital management and may offer similar outcomes at equal or lower costs. Available evidence suggests good feasibility and patient acceptance, although studies are limited by small sample sizes, short follow-up times and heterogeneous outcome measures. An optimal telehealth setting could combine structured telemonitoring of key parameters such as *P*_aCO_2__ and ventilator adherence with video consultations upon clinical deterioration, ideally tailored to patient complexity and initiated at home where feasible. Future research should evaluate such models prospectively, adopt standardised outcome measures and transparent reporting of economic perspectives, and generate the evidence base needed to support sustainable implementation across health systems.

## Supplementary material

**Please note:** supplementary material is not edited by the Editorial Office, and is uploaded as it has been supplied by the author. Table S1 00283-2026.SUPPLEMENT Table S2 00283-2026.SUPPLEMENT2 Table S3 00283-2026.SUPPLEMENT3 Figure S1 00283-2026.SUPPLEMENT4 AMSTAR 2 questionnaire 00283-2026.SUPPLEMENT5
