# Supplementary material for: Clinical and economic effectiveness of telehealth in home mechanical ventilation
Source: ERJ Open Res. 2026 Sep 28;12(5):00283-2026. doi: 10.1183/23120541.00283-2026 (PMC13617588; doi:10.1183/23120541.00283-2026)
Supplement: Supplementary file 1 [file 00283-2026.SUPPLEMENT.pdf]

**Additional File 1**

Table S-1: Search strategy used for the identification of relevant studies.

| Search (number) | Query |
|---|---|
| 1 | (effect* or effica* or cost-effect* or economic* or cost* or (clinc* adj2 outcome*) or outcome* or (treatment adj2 outcome)).ab,kf,ti,tw. |
| 2 | Cost-Effectiveness Analysis/ or Treatment Outcome/ or Cost Analysis/ or Cost-Benefit Analysis/ or Health Care Costs/ |
| 3 | 1 or 2 |
| 4 | Tele-Care or Tele-Referral or Telecare or Telehealth or Virtual Medicine or eHealth or mHealth or telemonitor* or tm or e-health or m-health or th or home-monitor* or (home adj2 monitor*) or (virtual adj2 medicine) or (remote adj2 monitoring) or (home adj2 telemonitor*) or telemedicine or tele-assist* or telehealth-support* or (tele adj2 health) or (mobile adj2 health) or digital health or (monitor* adj2 home)).ab,kf,ti. |
| 5 | Telemedicine/ or Remote Patient Monitoring/ or Digital Health/ or Remote Consultation/ |
| 6 | 4 or 5 |
| 7 | ((home adj5 ventilat*) or (outpatient adj2 ventilat*) or (domicil* adj5 ventilat*) or (ventilat* adj5 home) or (home adj2 niv) or (home adj2 non-invasive adj1 ventilat*) or (home adj2 ventilator-dependent) or (in-home adj2 ventilat*) or nihmv or (home adj2 invasive adj1 ventilat*) or (home adj2 mechanical* adj5 ventilat*) or home-ventilat*).ab,kf,ti,tw. |
| 8 | 3 and 6 and 7 |
