# Supplementary material for: Clinical and economic effectiveness of telehealth in home mechanical ventilation
Source: ERJ Open Res. 2026 Sep 28;12(5):00283-2026. doi: 10.1183/23120541.00283-2026 (PMC13617588; doi:10.1183/23120541.00283-2026)
Supplement: Supplementary file 3 [file 00283-2026.SUPPLEMENT3.pdf]

Additional File 3

Table S-3: Risk of bias assessment using ROBINS-I tool for non-randomized studies.

Study	Confounding	Participant selection	Classification of intervention	Deviation from intended intervention	Missing data	Measurement of outcomes	Selection of reported results	Overall bias
Vitacca et al., 2016 (18)	Moderate	Moderate	Low	Low	Low	Moderate	Low	Moderate
Le Mao et al., 2024 (24)	Moderate	Moderate	Low	Low	Low	Low	Low	Moderate
