# Supplementary material for: Clinical and economic effectiveness of telehealth in home mechanical ventilation
Source: ERJ Open Res. 2026 Sep 28;12(5):00283-2026. doi: 10.1183/23120541.00283-2026 (PMC13617588; doi:10.1183/23120541.00283-2026)

Additional File 4

Figure S-1: Risk of bias assessment using the Cochrane Risk of Bias 2 (RoB 2) tool.

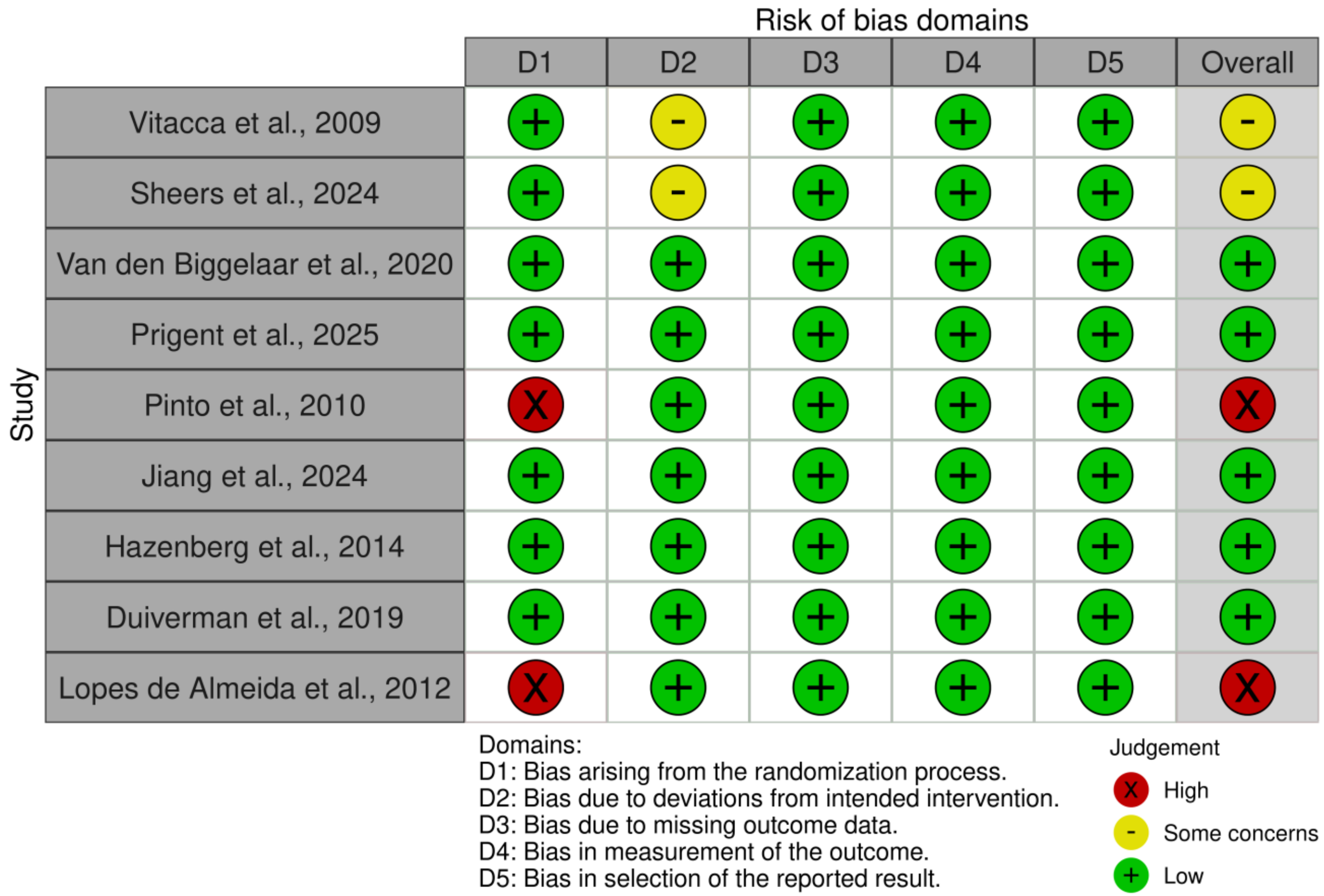

Supplement: Supplementary file 4 [file 00283-2026.SUPPLEMENT4.pdf]
